# Performance Assessment of the BluePoint MycoID *Plus* Kit for Identification of *Mycobacterium tuberculosis*, Including Rifampin- and Isoniazid-resistant Isolates, and Nontuberculous Mycobacteria

**DOI:** 10.1371/journal.pone.0125016

**Published:** 2015-05-04

**Authors:** Jung-Yien Chien, Tsung-Chain Chang, Wei-Yih Chiu, Chong-Jen Yu, Po-Ren Hsueh

**Affiliations:** 1 Graduate Institute of Clinical Medicine, National Taiwan University College of Medicine, Taipei, Taiwan; 2 Department of Internal Medicine, National Taiwan University Hospital, National Taiwan University College of Medicine, Taipei, Taiwan; 3 Chest Hospital, Ministry of Health and Welfare, Tainan, Taiwan; 4 Department of Medical Laboratory Science and Biotechnology, College of Medicine, National Cheng Kung University, Tainan, Taiwan; 5 Department of Laboratory Medicine, National Taiwan Udniversity Hospital, National Taiwan University College of Medicine, Taipei, Taiwan; The University of Hong Kong, CHINA

## Abstract

The performance of the BluePoint MycoID *plus* kit (Bio Concept Corporation, Taichung, Taiwan), which was designed to simultaneously detect *Mycobacterium tuberculosis* (MTB), rifampin- and isoniazid-resistant MTB, and nontuberculous mycobacteria (NTM) was first evaluated with 950 consecutive positive cultures in *Mycobacterium* Growth Indicator Tube (MGIT) system (BACTEC, MGIT 960 system, Becton-Dickinson, Sparks) from clinical respiratory specimens. The discrepant results between kit and culture-based identification were finally assessed by 16S rRNA gene sequencing and clinical diagnosis. The accuracy rate of this kit for identification of all *Mycobacterium* species was 96.3% (905/940). For MTB identification, the sensitivity, specificity, positive predictive value (PPV) and negative predictive value (NPV) of the kit were 99.7%, 99.3%, 99.0% and 99.8%, respectively. For rifampicin-resistant MTB identification, the sensitivity, specificity, PPV, and NPV of the kit were 100.0%, 99.4%, 91.3%, and 100.0%, respectively, while the corresponding values of isoniazid-resistant MTB identification were 82.6%, 99.4%, 95.0%, and 97.6%, respectively. In identifying specific NTM species, the kit correctly identified 99.3% of *M*. *abscessus* (147/148) complex, 100% of *M*. *fortuitum* (32/32), *M*. *gordonae* (38/38), *M*. *avium* (39/39), *M*. *intracellulare* (90/90), *M*. *kansasii* (36/36), and *M*. *avium* complex species other than *M*. *avium* and *M*. *intracellulare* (94/94). In conclusions, the diagnostic value of the BluePoint MycoID *plus* kit was superior to culture method for recoveries and identification of NTM to species level. In addition, the diagnostic accuracy of BluePoint MycoID *plus* kit in MTB identification was similar to conventional culture method with high accuracy rate of rifampicin-resistant *M*. *tuberculosis* identification.

## Introduction

The genus *Mycobacterium* comprises many species, including those in *Mycobacterium tuberculosis* (MTB) complex (MTBC) and nontuberculous mycobacteria (NTM). MTB infection leads to tuberculosis (TB) and remains one of the deadliest diseases worldwide [1]. Successful control of TB depends on rapid detection of MTB to prevent transmission. The conventional method for mycobacteria detection is based on acid-fast staining and culture. Staining have low sensitivity and does not discriminate MTB from NTM. A combination of solid and liquid cultures has acceptable sensitivity and the time to positivity for mycobacteria detection can be largely reduced to about 10 days by using the BACTEC *Mycobacterium* Growth Indicator Tube (MGIT) system (BACTEC, MGIT 960 system, Becton-Dickinson, Sparks, USA) [2]. However, the turn-around time of following identification steps and susceptibility test by conventional culture and biochemical methods still needs considerable times ranging from several days to weeks.

NTM are environmental microorganisms that are ubiquitous in soil and water. The incidence of infection due to NTM, such as pulmonary, soft tissue, bone, bloodstream, and central nervous system infections, has markedly increased over the past few decades [[3–9]. Species identification of NTM is recommended because different NTM species have different clinical presentations and drug-resistant patterns [3].

Therefore, new diagnostic methods that provide quick and specific results for species identification of mycobacteria and drug susceptibility of MTB among MGIT-positive samples will be extremely useful. Recently, the Bio Concept Corporation in Taiwan developed a membrane array (BluePoint MycoID *plus* kit) capable detection of two NTM complexes (*M*. *avium* complex [MAC], *M*. *abscessus* complex), 18 NTM species/groups (*M*. *avium*, *M*. *chelonae*, *M*. *fortuitum*, *M*. *gastri*, *M*. *gordonae*, *M*. *haemophilum*, *M*. *intracellulare*, *M*. *kansasii*, *M*. *marinum/M*. *ulcerans*, *M*. *malmoense*, *M*. *nonchromogenicum*, *M*. *peregrinum*, *M*. *scrofulaceum*, *M*. *simiae/M*. *lentiflavum*, *M*. *szulgai*, *M*. *terrae*, *M*. *xenopi* and *M*. *smegmatis* group [*M*. *smegmatis* and *M*. *goodie*]) and MTBC (including MTB and *M*. *bovis*), as well as 26 rifampicin- and isoniazid-resistance associated mutations in three genes (*rpoB*, *katG* and *inhA*) among MTB. The technique is based on reverse hybridization of polymerase chain reaction (PCR) products with an oligonucleotide membrane array. The target genes of the kit are the internal transcribed spacer (ITS) region between the genes encoding the ribosomal subunits 16S rRNA and the 23S rRNAs and the gene encoding the subunit B of DNA gyrase (*gyrB*). This was the first study to evaluate the performances of this kit in large sample size and simultaneously compared the accuracy of identification of MTB, rifampicin- or isoniazid-resistant MTB and species of NTM in positive MGIT cultures from clinical respiratory specimen; the results were compared with gold standards by a combination of routine culture-based identification, 16S rRNA gene sequencing, and clinical diagnosis.

## Methods

### Clinical Specimens and Cultures

During September to November 2013, a total of 950 positive MGIT cultures were collected, including 936 sputum and 14 bronchial wash specimens from 589 patients. All respiratory specimens were decontaminated and liquefied by adding an equal volume of NaOH–citrate–N-acetyl-L-cysteine at room temperature for 15 min and concentrated. The sediments were re-suspended in a minimal amount of phosphate buffer. The smears were examined using the Ziehl-Nielsen stain and graded according to the American Thoracic Society guidelines. Processed samples were inoculated into two types of media: the BACTEC MGIT tube (Becton-Dickinson, Sparks, USA) and the Lowenstein-Jensen Medium slant (Becton-Dickinson, Sparks, USA). An immumno-chromatographic assay using mouse monoclonal antibodies to detect MPT64 protein which is specific for *M*. *tuberculosis* complex was used in positive MGIT 960 cultures to distinguish MTB and NTM (SD TB Ag MPT 64 Rapid, Standard Diagnostics, Inc., Korea). Positive cultures by MGIT tube were subcultured on 7H11 plates (Becton-Dickinson, Sparks, USA) and identified by a combination of morphology, growth rate of the colonies, biochemical tests, including arylsulfatase reactions, Tween80 hydrolysis, urease test, and tolerance to 5% NaCl, and susceptibilities to cefoxitin and polymyxin B [10].

### Antimicrobial Susceptibility

The susceptibility testing of MTB complex was performed by indirect agar proportion method [11]. The critical concentrations were 0.2 (low-level) and 1.0 μg/mL (high-level) for isoniazid, 1.0 μg/mL for rifampicin, and 5.0 μg/mL for ethambutol according to the World Health Organization (WHO) recommendations [[11]. This study was approved by the Institutional Review Board of the National Taiwan University Hospital (201307051RIND).

### BluePoint MycoID Plus Kit

**Fig 1:**
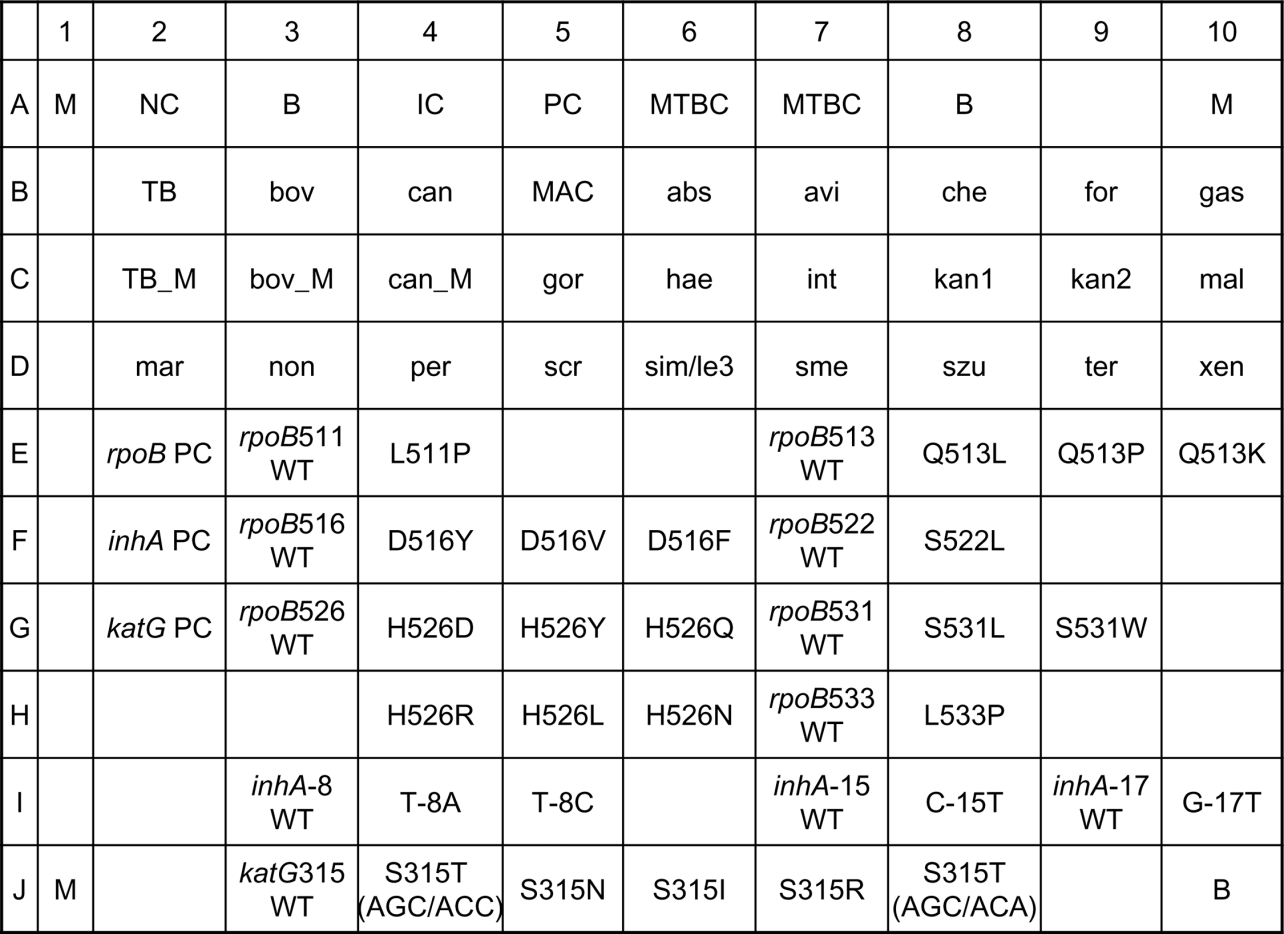
Layout of BluePoint MycoID *plus* kit for identification of nontuberculous mycobacteria species, *M*. *tuberculosis* and resistance-associated mutations. B, baseline; IC, internal amplification control; M, position marker; NC, negative control; PC, positive control; WT, wild-type; MTBC, *M*. *tuberculosis* complex; TB, *M*. *tuberculosis*; bov, *M*. *bovis*; can, *M*. *canettii*; MAC, *M*. *avium* complex; abs, *M*. *abscessus* complex; avi, *M*. *avium*; che, *M*. *chelonae*; for, *M*. *fortuitum*; gas, *M*. *gastri*; gor, *M*. *gordonae*; hae, *M*. *haemophilum*; int, *M*. *intracellulare*; kan, *M*. *kansasii*; mal, *M*. *malmoense*; mar, *M*. *marinum*; non, *M*. *nonchromogenicum*; per, *M*. *peregrinum*; scr, *M*. *scrofulaceum*; sim/le3, *M*. *simiae/M*. *lentiflavum*; sme, *M*. *smegmatis* group; szu, *M*. *szulgai*; ter, *M*. *terrae*; xen, *M*. *xenopi*. (*M*. *avium* complex includes *M*. *avium* subsp. *avium*, *M*. *avium* subsp. *paratuberculosis*, *M*. *avium* subsp. *hominissuis*, *M*. *avium* subsp. *silvaticum*, *M*. *lepraemurium*, *M*. *intracellulare*, *M*. *marseillense* sp. nov., *M*. *timonense* sp. nov., *M*. *bouchedurhonense* sp. nov., and *M*. *yongonense* sp. nov. *M*. *abscessus* complex includes *M*. *abscessus*, *M*. *massiliense*, and *M*. *bolletii*. *M*. *smegmatis* group includes *M*. *smegmatis* and *M*. *goodie*.).

**Fig 2:**
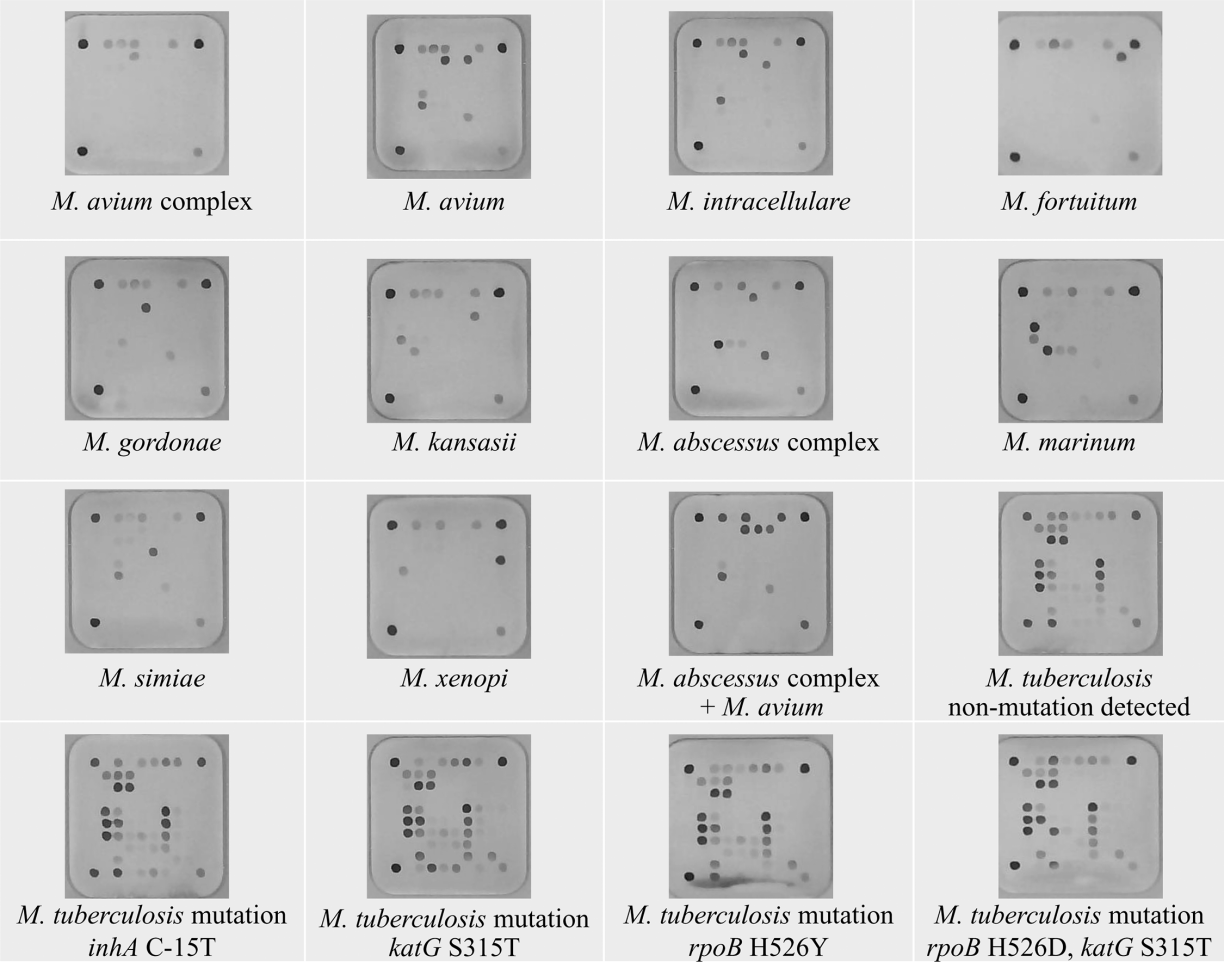
The performances of selected mycobacterial species for identification and resistance-associated mutations for isoniazid (*inhA* and *katG*) and rifampin (*rpoB*) of *M*. *tuberculosis* isolates by the BluePoint MycoID *plus* kit among the positive cultures in *Mycobacterium* Growth Indicator Tubes.

DNA was extracted from a positive MGIT culture by the boiling method and the test was done according to the instructions supplied by the manufacturer as previously described [12]]. The layout and designations of oligonucleotide probes and targets for the probes are illustrated in Figs 1 and 2. The test procedure consisted of amplification of the ITS and *gyrB* regions by a multiplex PCR, hybridization of the digoxigenin-labeled amplicons with the array, and reaction with enzyme-conjugated anti-digoxigenin antibodies. The hybridized spot was read by a simple reader supplied by the kit manufacturer. A strain was identified to the species level when a species-specific probe and the positive control probe were simultaneously hybridized. If only the positive control probe was hybridized, the microorganism was identified to the genus level (*Mycobacterium* species).

## Discrepant Analysis

The results of mycobacterial species identification by the BluePoint MycoID *plus* kit and by the culture method were initially evaluated and compared. When there was a difference in the species identification results obtained by the kit and by the culture method, 16S rRNA sequencing analysis was used for further species identification [13]. Sequencing analysis of the 16S rRNA gene was performed using two primers F(5’-GAAGAGTTTGATCMTGGCTC-3’ and R(5’-GCGTGGACTACCAGGGTATC-3’) and the resulting sequence was used for a BLAST search [14]. If a discrepant identification was a strain of MTB, medical records, including history, medical conditions, radiology, microbiology results, treatment course, physician prescription, and follow-up observations were reviewed to perform the assessment which served as the final diagnosis. Two categories of samples were considered true-positives for MTB as previously described: (i) samples that were culture positive for MTB and (ii) samples that were culture negative for MTB but originated from a patient whose other samples within 3 days were culture positive for MTB [15]. When there was a difference in the drug resistance results by the kit and agar proportional method, the corresponding resistance-associated mutations were sequencing with specific primers, *rpoB* F(5’-CCATCGAATATCTGGTCCGC-3’) / R(5’-CGCATCGATCGGCGAAT-3’), *inhA* F(5’-CATCGACACCGATATGACCC-3’) / R(5’-CGACCGTCATCCAGTTGTAG-3’) and *katG* F(5’-AATTCCTCGGGGTGTTCCA-3’) / R(5’-GAACGGCAACCCGGAC-3’).

### Statistical Analysis

Statistical comparisons were calculated by the chi-square test or Fisher’s exact test, where appropriate. All tests were two-sided. A *P* value <0.05 was considered to represent statistical significance.

## Results

Of those 950 positive MGIT samples, 935 (557 NTM and 378 MTB) and 983 (597 NTM and 386 MTB) mycobacterial isolates were identified by culture method and BluePoint MycoID *plus* kit, respectively (Table 1, *P*<0.001). The most prevalent species were MTB, followed by MAC, *M*. *abscessus* complex, *M*. *fortuitum*, *M*. *gordonae* and *M*. *kansasii*. A total of 42 (4.4%) specimens with mixed species were found by the kit but none was found by culture method. Among them, 7 were mixed species of MTB and NTM (6 samples contained MTB and one NTM species and one sample contained MTB and two NTM species), while the remaining 35 contained 2 NTM species. A total of 16 different species/complexes were identified by the BluePoint MycoID *plus* kit, while only 8 different species/complexes were identified by culture method (Table 1).

**Table 1:** Results obtained by culture method and the BluePoint MycoID *plus* kit from 950 positive Mycobacteria Growth Indicator Tubes (MGIT).

Species or complexes	No. (%)		
	Culture method	BluePoint MycoID *plus* kit	Final identification
*M*. *tuberculosis*, non-MDR	363 (38.8)	372 (37.8)	368 (39.1)
*M*. *tuberculosis*, MDR	15 (1.6)	14 (1.4)	15 (1.6)
*M*. *abscessus complex*	149 (15.9)	152 (15.5)	148 (15.7)
MAC	232 (24.8)	101 (10.3)	94 (10.0)
*M*. *intracellulare*	0 (0.0)	93 (9.5)	90 (9.6)
*M*. *avium*	0 (0.0)	46 (4.7)	39 (4.1)
*M*. *fortuitum*	39 (4.2)	40 (4.1)	32 (3.4)
*M*. *kansasii*	33 (3.5)	39 (4.0)	36 (3.8)
*M*. *gordonae*	37 (4.0)	42 (4.3)	38 (4.0)
*M*. *simiae/M*. *lentiflavum*	0 (0.0)	19 (1.9)	15 (1.6)
*M*. *scrofulaceum*	5 (0.5)	5 (0.5)	4 (0.4)
*M*. *chelonae*	1 (0.1)	1 (0.1)	1 (0.1)
*M*. *nonchromogenicum*	0 (0.0)	2 (0.2)	2 (0.2)
*M*. *gastri*	0 (0.0)	5 (0.5)	0 (0.0)
*M*. *peregrinum*	0 (0.0)	4 (0.4)	0 (0.0)
*M*. *xenopi*	0 (0.0)	1 (0.1)	0 (0.0)
*M*. *smegmatis* group	0 (0.0)	1 (0.1)	0 (0.0)
*M*. *mageritense*	0 (0.0)	0 (0.0)	6 (0.6)
*M*. *senegalense*	0 (0.0)	0 (0.0)	5 (0.5)
*M*. *mantenii*	0 (0.0)	0 (0.0)	3 (0.3)
*M*. *interjectum*	0 (0.0)	0 (0.0)	3 (0.3)
*M*. *septicum*	0 (0.0)	0 (0.0)	3 (0.3)
*M*. *cosmeticum*	0 (0.0)	0 (0.0)	2 (0.2)
*M*. *neoaurum*	0 (0.0)	0 (0.0)	1 (0.1)
*M*. *llatzerense*	0 (0.0)	0 (0.0)	1 (0.1)
*M*. *porcinum*	0 (0.0)	0 (0.0)	1 (0.1)
*M*. *canariasense*	0 (0.0)	0 (0.0)	1 (0.1)
*M*. *moriokaense*	0 (0.0)	0 (0.0)	1 (0.1)
*M*. *shimoidei*	0 (0.0)	0 (0.0)	1 (0.1)
*M*. *longobardum*	0 (0.0)	0 (0.0)	1 (0.1)
*M*. *timonense*	0 (0.0)	0 (0.0)	1 (0.1)
*Mycobacterium* species	61 (6.5)	46 (4.7)	28 (3.0)
Total	935	983	940 (100)

**Note**

MAC: *M*. *avium* complex. MDR: multidrug-resistant.

A total of 71 discrepant results and one indeterminate identification were found between culture method and the BluePoint MycoID *plus* kit (Table 2). According to final identification based on 16S rRNA gene sequencing result and clinical findings, 36 (50.7%) isolates of 71 discrepant results was corrected identified to species level by BluePoint MycoID *plus* kit, but only 4 (5.6%, *P*<0.001) was corrected identified by culture method. In identifying specific NTM species, the BluePoint MycoID *plus* kit correctly identified 147 (99.3%) of 148 *M*. *abscessus* complex, 32 (100%) of 32 *M*. *fortuitum*, 38 (100%) of 38 *M*. *gordonae*, 39 (100%) of 39 *M*. *avium*, 90 (100%) of 90 *M*. *intracellulare*, 94 (100%) of 94 MAC and 36 (100%) of 36 *M*. *kansasii*. Altogether, among the 940 samples with final identification, BluePoint MycoID *plus* kit had higher accuracy rate of species identification than culture method (905/940, 96.3% v.s. 873/940, 92.9%; *P* = 0.001).

**Table 2:** Analysis of the discrepant results of species identification by culture method and the BluePoint MycoID *plus* kit.

Mycobacterial species or complexes identified by:				No.
Culture method	BluePoint MycoID *plus* kit^a^	16S rRNA gene sequencing	Final identification	
*M*. *abscessus* complex	*M*. *fortuitum*	*M*. *fortuitum*	*M*. *fortuitum*	1
	*Mycobacterium* species	*M*. *canariasense*	*M*. *canariasense*	1
		*M*. *mageritense*	*M*. *mageritense*	1
	***M*. *tuberculosis***	*M*. *abscessus*	*M*. *abscessus*	1
		*M*. *longobardum*	*M*. *longobardum*	1
		*M*. *senegalense*	*M*. *tuberculosis /M*. *senegalense*	1
	*M*. *fortuitum /M*. *intracellulare*	*M*. *fortuitum*	*M*. *fortuitum*	1
	*M*. *tuberculosis /M*. *fortuitum*	*M*. *fortuitum*	*M*. *tuberculosis /M*. *fortuitum*	1
*M*. *fortuitum*	*M*. *abscessus* complex	*M*. *abscessus*	*M*. *abscessus*	4
	**MAC**	*M*. *mageritense*	*M*. *mageritense*	1
	*Mycobacterium* species	*M*. *llatzerense*	*M*. *llatzerense*	1
		*M*. *mageritense*	*M*. *mageritense*	3
		*M*. *porcinum*	*M*. *porcinum*	1
		*M*. *senegalense*	*M*. *senegalense*	2
*M*. *kansasii*	*Mycobacterium* species	*M*. *lentiflavum*	*M*. *lentiflavum*	1
*M*. *scrofulaceum*	*Mycobacterium* species	*M*. *interjectum*	*M*. *interjectum*	1
MAC	*M*. *simiae (M*. *lentiflavum)*	*M*. *lentiflavum*	*M*. *lentiflavum*	1
		*M*. *simiae*	*M*. *simiae*	1
	*Mycobacterium* species	*M*. *cosmeticum*	*M*. *cosmeticum*	1
		*M*. *interjectum*	*M*. *interjectum*	2
		*M*. *mantenii*	*M*. *mantenii*	3
		*M*. *shimoidei*	*M*. *shimoidei*	1
		*M*. *timonense*	*M*. *timonense*	1
*Mycobacterium* species	*M*. *abscessus* complex	*M*. *abscessus*	*M*. *abscessus*	2
	***M*. *abscessus* complex**	*M*. *neoaurum*	*M*. *neoaurum*	1
	*M*. *avium*	*M*. *avium*	*M*. *avium*	1
	*M*. *fortuitum*	*M*. *fortuitum*	*M*. *fortuitum*	2
	***M*. *fortuitum***	*M*. *senegalense*	*M*. *senegalense*	2
	*M*. *gordonae*	*M*. *gordonae*	*M*. *gordonae*	1
	*M*. *kansasii*	*M*. *kansasii*	*M*. *kansasii*	3
	*M*. *nonchromogenicum*	*M*. *nonchromogenicum*	*M*. *nonchromogenicum*	1
	***M*. *peregrinum***	*M*. *moriokaense*	*M*. *moriokaense*	1
		*M*. *septicum*	*M*. *septicum*	2
	*M*. *simiae/M*. *lentiflavum*	*M*. *lentiflavum*	*M*. *lentiflavum*	11
		*M*. *simiae*	*M*. *simiae*	1
	***M*. *simiae/M*. *lentiflavu)***	*M*. *cosmeticum*	*M*. *cosmeticum*	1
		*M*. *mageritense*	*M*. *mageritense*	1
	***M*. *tuberculosis***	Failure	*Mycobacterium* species	1
	*M*. *nonchromogenicum /M*. *scrofulaceum*	*M*. *nonchromogenicum*	*M*. *nonchromogenicum*	1
	***M*. *gordonae /M*. *peregrinum***	*M*. *septicum*	*M*. *septicum*	1
	*M*. *gordonae /M*. *kansasii*	*M*. *kansasii*	*M*. *kansasii*	1
*M*. *tuberculosis*	***M*. *kansasii***	*M*. *tuberculosis*	*M*. *tuberculosis*	1
No mycobacteria detected	***M*. *tuberculosis*, non-MDR**	Failure	No mycobacteria detected	1
	*M*. *tuberculosis*, non-MDR	Failure	*M*. *tuberculosis*, non-MDR	3

**Note:** “a” means isolates marked in bold indicate discrepant results between the results from the BluePoint MycoID *plus* kit and final identification.

MAC: *M*. *avium* complex.

For MTB identification, the BluePoint MycoID *plus* kit produced 930 concordant (377 positive and 553 negative) and 10 discordant results with culture method (one was culture-positive/kit-negative and 9 were culture-negative/kit-positive, Table 3). Results of the analyses of the 10 specimens with discrepant results of MTB identification are shown in Table 4. Among the 9 culture-negative/kit-positive specimens, 5 were proved to be true positives by clinical evaluation (all 5 specimens came from patients with active tuberculosis without treatment; among that 5 samples, 3 specimens were contaminated by bacterium during subculture and 2 isolates were identified as *M*. *abscessus* complex by the culture method) and 4 were false positivity (all from patients with pulmonary tuberculosis who had been effectively treated and showed evidence of culture conversion at the time of specimen collection). After discrepant analysis, 383 samples were shown to be true positives for MTB (Table 3). As determined by the final identification, the sensitivity, specificity, positive predictive value (PPV) and negative predictive value (NPV) of the BluePoint MycoID *plus* kit for MTB identification were 99.7%, 99.3%, 99.0% and 99.8%, respectively. Similar results were obtained by the culture method (98.7%, 100.0%, 100.0% and 99.1%, respectively; Table 3). A total of 1 (0.3%) and 5 (1.3%) respiratory samples had a false-negative result and a total of 4 (0.7%) and 0 (0.0%) samples had a false-positive result by the BluePoint MycoID *plus* kit and the culture method, respectively. The difference between these two methods for MTB identification was not significant.

**Table 3:** Comparison of the culture method and BluePoint MycoID *plus* kit for identification of *M*. *tuberculosis* in positive Mycobacteria Growth Indicator Tubes (MGIT).

Assay	No. of samples				Performance % (95% confidence interval)			
					Sensitivity	Specificity	PPV	NPV
	Culture method							
	Positive		Negative					
BluePoint MycoID *plus* kit	Positive	Negative	Positive	Negative				
	377	1	9	553	99.7 (98.5–100.0)	98.4 (97.0–99.3)	97.7 (95.6–98.9)	99.8 (99.0–100.0)
	Final identification							
	Positive		Negative					
BluePoint MycoID *plus* kit	Positive	Negative	Positive	Negative				
	382	1	4	553	99.7 (98.6–100.0)	99.3 (98.2–99.8)	99.0 (97.3–99.7)	99.8 (99.0–100.0)
Culture method	Positive	Negative	Positive	Negative				
	378	5	0	557	98.7 (97.0–99.6)	100.0 (99.3–100.0)	100.0 (99.0–100.0)	99.1 (97.9–99.7)

**Note:** PPV: Positive predictive value. NPV: Negative predictive value.

**Table 4:** Characterizations of cases with discrepant identification of *M*. *tuberculosis* by culture method and the BluePoint MycoID *plus* kit

Case	Diagnosis	Smear grading^a^	Cavity in CXR^a^	Effective treatment	Culture conversion	Species identification		
						Culture method	BluePoint MycoID*plus* kit^b^	Final identification
1	Pulmonary TB	4+	+	NA	No	*M*. *abscessus*	*M*. *tuberculosis*	*M*. *tuberculosis* & *M*. *senegalense*
2	Pulmonary TB	-	-	NA	No	*M*. *abscessus*	*M*. *tuberculosis /M*. *fortuitum*	*M*. *tuberculosis/M*. *fortuitum*
3	Pulmonary TB	4+	+	NA	No	No mycobacteria detected	*M*. *tuberculosis*	*M*. *tuberculosis*
4	Pulmonary TB	4+	+	NA	No	No mycobacteria detected	*M*. *tuberculosis*	*M*. *tuberculosis*
5	Pulmonary TB	-	-	NA	No	No mycobacteria detected	*M*. *tuberculosis*	*M*. *tuberculosis*
6	Pulmonary TB	2+	+	2 months	Yes	*Mycobacterium* species	***M*. *tuberculosis***	*Mycobacterium* species
7	Pulmonary TB	4+	+	5 months	Yes	*M*. *abscessus*	***M*. *tuberculosis***	*M*. *longobardum*
8	Pulmonary TB	2+	+	5 months	Yes	*M*. *abscessus*	***M*. *tuberculosis***	*M*. *abscessus*
9	Pulmonary TB	2+	+	3 months	Yes	No mycobacteria detected	***M*. *tuberculosis***	No mycobacteria detected
10	Pulmonary TB	-	-	NA	No	*M*. *tuberculosis*	***M*. *kansasii***	*M*. *tuberculosis*

**Note:** “a” means the status of disease before starting anti-TB treatment. “b” means isolates marked in bold indicate discrepant results between the results from the BluePoint MycoID *plus* kit and final identification.

CXR: chest x-ray. NA: not applicable. TB: tuberculosis.

Among 378 MTB isolates, the accuracy rate for identifying multidrug-resistant (MDR) MTB was 93.3% (14/15, Table 1). All of 21 rifampicin-resistant MTB isolates were correctly identified by kit (Table 5). However, among the 23 rpoB mutation-positive isolates identified by the kit, 2 (8.7%) isolates had false-positive results (susceptible to rifampicin by the proportion method and no mutation being found by gene sequencing). Among 46 isoniazid-resistant MTB isolates (25 were high-level and 21 were low-level resistance), 38 (82.6%) were identified by kit. Among 5 high-level isoniazid-resistant isolates with no katG or inhA mutations detected by the kit, sequencing analysis revealed that 2 (40%) had katG gene mutations (G279D and I313T), mutations that are not targets of the kit. Among the 40 isolates with either katG (n = 24) or inhA gene (n = 21) mutation determined by the kit, 2 (5.0%) isolates had false-positive results (susceptible to isoniazid by the proportion method and no mutation being found by gene sequencing). As determined by agar proportion method, for rifampicin-resistant MTB identification, the sensitivity, specificity, PPV, and NPV of the kit were 100.0%, 99.4%, 91.3%, and 100.0%, respectively, while the corresponding values for isoniazid-resistance MTB identification were 82.6%, 99.4%, 95.0%, and 97.6%, respectively.

**Table 5:** Drug resistance by conventional agar proportion method and resistance-associated gene mutations detected by BluePoint MycoID *plus* kit

	**Drug susceptibility, No. (%)**				
	Rifampicin		Isoniazid		
Gene mutations	Resistant	Susceptible	High-level resistant	Low-level resistant	Susceptible
*rpoB*					
S522L	1 (4.8)	0 (0.0)			
H526D	3 (14.3)	0 (0.0)			
H526Y	0 (0.0)	2 (0.6)^a^			
H526Y, S531L	1 (4.8)	0 (0.0)			
S531L	16 (76.2)	0 (0.0)			
-	0 (0.0)	354 (99.4)			
*katG/ InhA*					
S315T/-			17 (68.0)	0 (0.0)	0 (0.0)
S315I/-			0 (0.0)	0 (0.0)	2 (0.6)^a^
S315I/ C-15T			0 (0.0)	4 (19.0)	0 (0.0)
S315I/ G-17T			0 (0.0)	1 (4.8)	0 (0.0)
-/ C-15T			3 (12.0)	12 (57.1)	0 (0.0)
-/ G-17T			0 (0.0)	1 (4.8)	0 (0.0)
-/-			5 (20.0)^b^	3 (14.3)	329 (99.4)

**Note:** “a” means no of mutations was detected by sequencing analysis.”b” means both *katG* G279D and *katG* I313T mutations were found in 2 isolates by gene sequencin

## Discussion

Comparing with subculture method from positive MGIT tubes, we found BluePoint MycoID *plus* kit had the advantages of better recoveries and accuracy rate of NTM species identification (92.9% v.s. 96.3%, respectively; *P* = 0.001). For MTB identification, the performance of kit tendered to be more sensitive but less specificity than conventional subculture method, with a 100% and 82.6% sensitivity for rifampicin- and isoniazid-resistant MTB identification.

Physicians should always consider NTM as a possible pathogen causing an infection because the risk of a positive culture for NTM is increasing worldwide [[4,16–18]. We found that the number of mixed cultures detected by the kit (42 samples, 4.4%) was higher than that obtained by the culture method (0 samples). The mixed culture rate (4.4%) was less than that (12%) reported by Shenai et al [19], but similar to that (3.2%) reported by Lu et al [12]. Because conventional culture techniques are based on isolation followed by colony identification, they tend to isolate a single species of *Mycobacterium* with a more rapid growth rate or a dominant cell number in a sample and tend to underestimate the number of isolates among specimens containing more than one *Mycobacterium* species [13].

The major difference between MTB and NTM is that the former can spread via person to person contact. It is particularly important to detect MTB in clinical specimens as early as possible to interrupt the dissemination and transmission of the organism. For detection of MTB, we found the kit tended to have fewer false-negative results than culture method (0.3% v.s. 1.3%). In the current study, the false-negative results of immumno-chromatographic assay by mpt64 detection and bacterial contamination on subculture plates were the most likely reason for the majority (60%) of false-negative results from the culture method. Other possible reasons might include the small number of MTB isolates from patients with low bacterial burden (such as low staining grade), a suboptimal target extraction, or unequal distribution in the test suspension. In contrast, PCR-based assays can yield more false-positive results than culture methods if specimens from patients under effective anti-TB treatment contain nonviable mycobacteria [20].

Early identification of drug resistance is also crucial because a MTB isolate resistant to one drug, especially isoniazid and rifampin, is more likely to be resistant to other anti-TB drugs [21]. Thus there is a great need to rapidly determine the susceptibility patterns of MTB strains. Over 95% strains with resistance to rifampicin are associated with mutations within an 81-bp rifampicin resistance-determining region (RRDR) of the *rpoB* gene, corresponding to codons 506–533 [22]]. We found that the BluePoint MycoID *plus* kit was able to detect all rifampicin-resistant MTB isolates with only 2 (8.7%) false-positive results, giving a good sensitivity (100.0%), specificity (99.4%), PPV (91.3%) and NPV (100.0%) for rifampicin-resistant MTB identification. Isoniazid resistance is more genetically heterogeneous and may involve mutations in the *katG* gene or the *inhA* gene or both [22]. While mutation alternations in the promoter of *inhA* are associated with low-level resistance to isoniazid, in the *katG* gene, they confer moderate- to high-level drug resistance [23]. However, about 10%-20% isoniazid-resistant strains did not have known mutations in either *katG* or *inhA* genes [24]. The BluePoint MycoID *plus* kit did not identify 17.4% (8/46) of isoniazid-resistant MTB isolates, resulting in a sensitivity of 82.6% to identify isoniazid-resistant MTB isolates. Consequently, 6.7% (1/15) of MDR MTB isolates were not identified by this kit (Table 1).

Matrix-assisted laser desorption/ionization time-of-flight mass spectrometry (MALDI-TOF MS) is now used in laboratories to rapidly identify bacteria [25], including *Mycobacterium* species [26–28]. However, Lotz et al found that although MALDI-TOF MS can correctly identify 97% of *Mycobacterium* species from Lowenstein-Jensen media, the results from MGIT medium (about 77% accuracy rate) were not as good as those obtained from solid medium [26]. This was probably because of spectral acquisition failures, due either to the low number of bacteria or to potential interference of the supplements included in the complex medium [26].

Currently, two commercialized line-probe assay also has a capability to identify mycobacteria in liquid cultures. The GenoType Mycobacterium CM assay (Hain Lifescience GmbH, Germany) can detect MTBC and 24 of the most common NTM species (additional 19 species by their AS assay) and the INNO-LiPA MYCOBACTERIA v2 kit (Fujirebio Europe N.V.) can detect MTBC and 16 NTM species. However, due to the banding patterns of the line-probe assay are not always obvious, a well-trained medical technician is needed to interpret the results [29]. For detecting rifampin resistance, the current kit consists of 8 wild-type and 17 mutant *rpoB* probes to detect mutations in 6 codons in the hot-spot region of the *rpoB* gene. In addition, for detecting isoniazid resistance, the current kit uses one wild-type and five mutant *katG* probes and two wild-type and three mutant *inhA* probes to detect mutations in codon 315 of the *katG* gene and the upstream regulatory region (nucleotide positions -8 and -15) of the *inhA* gene. A line-probe assay, the GenoType MTBDR*plus* test (Hain Lifescience GmbH, Nehren, Germany) also can identify the MTBC and detect resistance to rifampin and isoniazid. However, certain mutations, such as *rpoB* H526Q, do not have a corresponding probe, so a mutant harbouring this mutation cannot be clearly identified by the GenoType MTBDR*plus* test. The current kit has the advantage of low cost and its hybridization patterns can be clearly interpreted. It can also identify more exact nucleotide substitutions in the mutated codons, which is useful for epidemiological investigations.

The major flaw of the BluePoint MycoID *plus* kit is its limited ability to correctly identify rare NTM species, such as *M*. *canariasense*, *M*. *cosmeticum*, *M*. *interjectum*, *M*. *llatzerense*, *M*. *longobardum*, *M*. *mageritense*, *M*. *mantenii*, *M*. *moriokaense*, *M*. *neoaurum*, *M*. *porcinum*, *M*. *senegalense*, *M*. *septicum*, *M*. *shimoidei* and *M*. *timonense*, as these mycobacteria are not target species of the kit. This chip also failed to differentiate between *M*. *marinum* and *M*. *ulcerans*, as well as between *M*. *simiae* and *M*. *lentiflavum*, which are very rare causes of lung disease. The kit was unable to detect all resistance-conferring mutations, similarly to previous finding in laboratory drug-resistant strains [29]. In addition, the false reaction of rifampicin and isoniazid resistant mutations might be cause by heteroresistance of the bacilli. Previous studies had found heterogeneous peaks sequencing could be found in up to 30% of the clinical isolates [30]. Such heteroresistance might refer to a small minority of mutant bacilli coexist in a clinical sample with wild-type bacilli. This high-sensitive array [29] could detect the mutant bacilli, but these mutant fractions were so small that both conventional culture and genes sequencing techniques detected only the majority non-mutant bacilli.

In summary, the increasing incidence of drug-resistant TB and NTM has prompted the need for sensitive, fast, and accurate methods to discriminate NTM from MTB and determine resistance pattern of MTB. Our results showed that the diagnostic value of the BluePoint MycoID *plus* kit was superior to the conventional culture method for recoveries and identification of NTM to species level. In addition, the diagnostic accuracy of BluePoint MycoID *plus* kit in MTB identification was similar to that of the conventional culture method, and had a high accuracy rate for rifampicin-resistant MTB identification.
